# Carotid artery velocity time integral and corrected flow time measured by a wearable Doppler ultrasound detect stroke volume rise from simulated hemorrhage to transfusion

**DOI:** 10.1186/s13104-021-05896-y

**Published:** 2022-01-10

**Authors:** Jon-Émile S. Kenny, Igor Barjaktarevic, David C. Mackenzie, Mai Elfarnawany, Zhen Yang, Andrew M. Eibl, Joseph K. Eibl, Chul-Ho Kim, Bruce D. Johnson

**Affiliations:** 1 Health Sciences North Research Institute, 56 Walford Rd, Sudbury, ON P3E 2H2 Canada; 2 Flosonics Medical, 325 Front Street, Toronto, ON M5V 2Y1 Canada; 3 Division of Pulmonary and Critical Care, Department of Medicine, David Geffen School of Medicine at UCLA, Los Angeles, CA USA; 4 Department of Emergency Medicine, Maine Medical Center, Portland, ME USA; 5 Tufts University School of Medicine, Boston, MA USA; 6 Northern Ontario School of Medicine, Sudbury, ON Canada; 7 Human Integrative and Environmental Physiology Laboratory, Department of Cardiovascular Diseases, Mayo Clinic, Rochester, MN USA

**Keywords:** Carotid Doppler, Stroke volume, Velocity time integral, Corrected flow time

## Abstract

**Objective:**

Doppler ultrasonography of the common carotid artery is used to infer stroke volume change and a wearable Doppler ultrasound has been designed to improve this workflow. Previously, in a human model of hemorrhage and resuscitation comprising approximately 50,000 cardiac cycles, we found a strong, linear correlation between changing stroke volume, and measures from the carotid Doppler signal, however, optimal Doppler thresholds for detecting a 10% stroke volume change were not reported. In this *Research Note*, we present these thresholds, their sensitivities, specificities and areas under their receiver operator curves (AUROC).

**Results:**

Augmentation of carotid artery maximum velocity time integral and corrected flowtime by 18% and 4%, respectively, accurately captured 10% stroke volume rise. The sensitivity and specificity for these thresholds were identical at 89% and 100%. These data are similar to previous investigations in healthy volunteers monitored by the wearable ultrasound.

## Introduction

Inferring change in stroke volume (SV) is the bedrock of functional hemodynamic monitoring [1, 2]. Yet measuring SV change (SV_∆_) is clinically challenging, so surrogates like the common carotid artery Doppler pulse have been proposed [3, 4] with some conflicting data [5]. We contend that statistically-inadequate beat sampling coupled with variation introduced by the respiratory cycle are important arbiters of inconsistent clinical research [6]. To rectify the shortcomings of handheld examinations, we developed a wireless, wearable Doppler ultrasound [7, 8]. This device adheres to the neck, maintains a constant insonation angle and accurately measures beat-to-beat changes across multiple cardiorespiratory cycles [6, 7].

In early, proof-of-concept investigations, the carotid artery maximum velocity time integral (VTI) and corrected flow time (ccFT) accurately identified a + 10% SV_∆_ with thresholds of + 15% and + 2–4%, respectively [9, 10]. More recently, in a human model of hemorrhage and resuscitation comprising approximately 50,000 cardiac cycles, we found a strong, linear correlation between SV_∆_, and both changing maximum carotid VTI (VTI_∆_) and ccFT (ccFT_∆_) [8]. While 10% SV_∆_ associated with 18% VTI_∆_ and 4.3% ccFT_∆_, the *optimal* VTI_∆_ and ccFT_∆_ thresholds for detecting a + 10% SV_∆_ were not investigated. In this *Research Note*, we report these thresholds, their sensitivities, specificities and areas under their receiver operator curves (AUROC).

## Main text

### Methods

#### Clinical setting

11 healthy, adult volunteers with no cardiovascular history and who provided written, informed consent were recruited. The study was approved by the Research Ethics Board of the Mayo Clinic (IRB number 19–010,136).

#### Adherent Doppler system

**Fig. 1:**
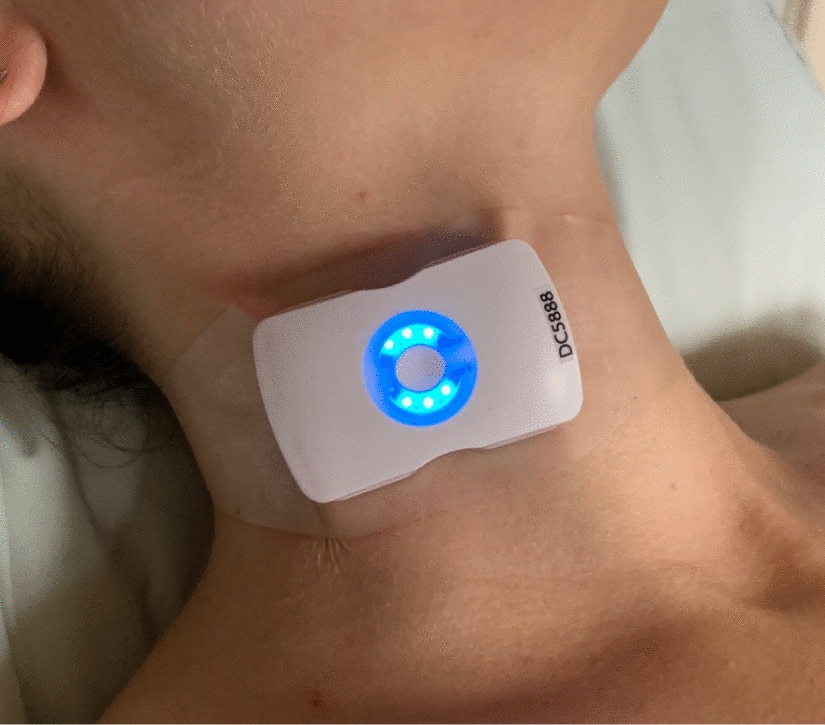
Picture of wireless, wearable Doppler ultrasound device

The U.S. Food and Drug Administration (FDA) cleared, 4 MHz Doppler ultrasound (Fig. 1) (Flosonics Medical, Sudbury, Canada) was placed and the ccFT, maximum and power-weighted, i.e., centroid, VTIs were captured [6, 8–10].

#### Lower body negative pressure (LBNP) and stroke volume

As previously reported [6], all subjects underwent a 7-stage protocol in duplicate; a non-invasive SV monitor, the Nexfin® (Edwards Lifesciences, Irvine, California), was synchronized with the Doppler monitor for each cardiac cycle.

#### Statistical analysis

All data was averaged over 10-s windows. SV and carotid Doppler were referenced to resting baseline to model hemorrhage. Cardiac cycles with artifact or during LBNP stage transition were excluded, as described [8]. SV change from the lowest-tolerated LBNP stage back to atmosphere modeled rapid blood transfusion, data from this transition was included.

Each 10-s data point was dichotomized as ≥  + 10% SV_∆_ or <  + 10% SV_∆_ based on the non-invasive SV monitor. An equal number of data points were randomly sampled from negative pressure stages without replacement to match the sample size of the positive cases (≥ + 10% SV_∆_) for 1000 iterations. Within each iteration, the optimal threshold for each metric was selected using the Youden index. Averages of optimal thresholds and the corresponding sensitivities and specificities were calculated from all iterations and reported as the best final estimations. AUROC was calculated using the subsamples that produced the same optimal thresholds as the final estimation after 1000 iterations.

### Results

**Fig. 2:**
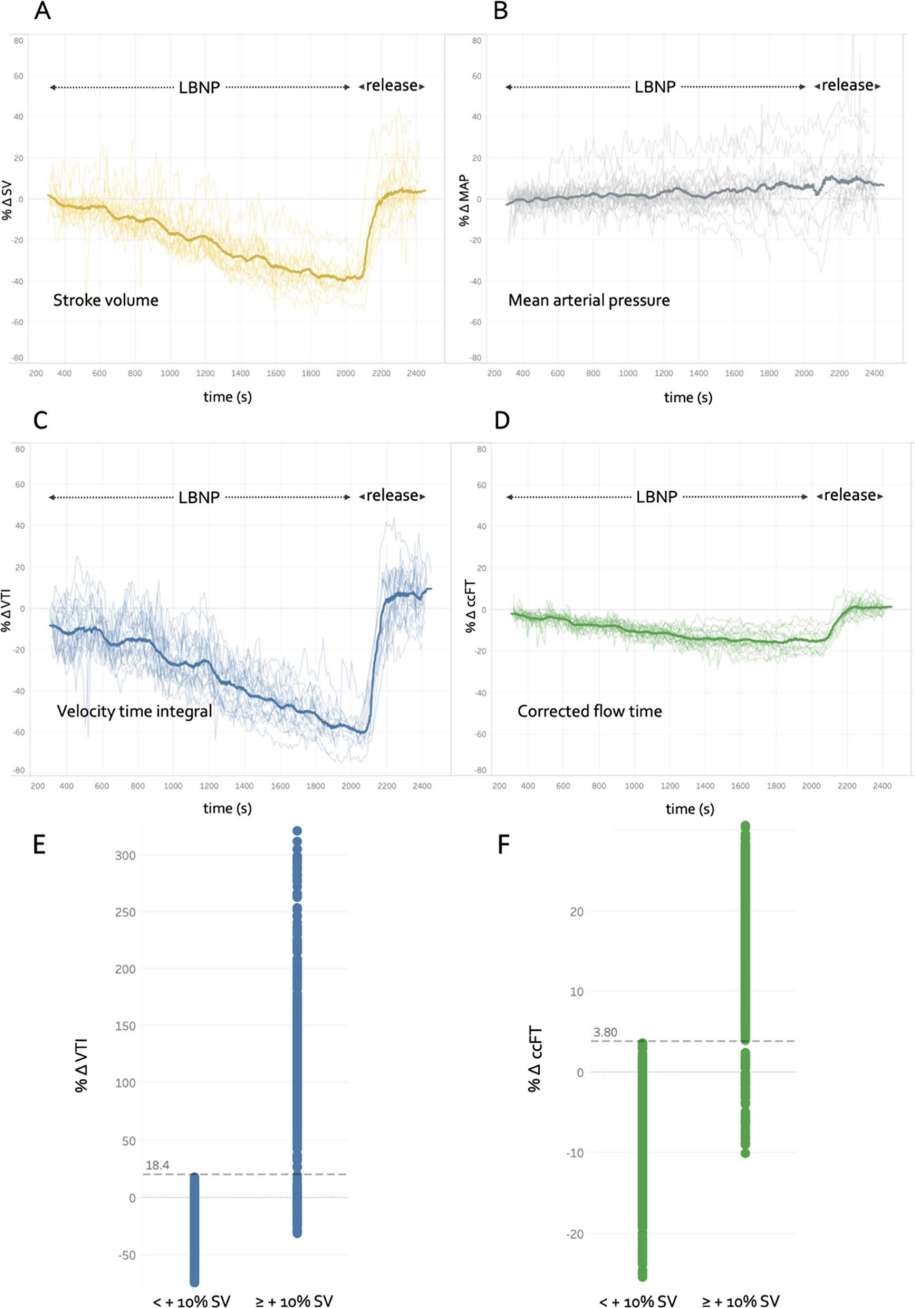
Hemodynamic data captured during lower body negative pressure (LBNP) and release. Measures from **A**–**D** are synchronously captured. Each faint line represents a single protocol, while the emboldened line represents the average of all protocols. **A** Stroke volume (SV) percent change during progressively severe LBNP (i.e., hemorrhage model) and release of LBNP (i.e., rapid transfusion model). **B** Mean arterial pressure (MAP) percent change. **C** velocity time integral (VTI) from the wearable Doppler percent change. **D** corrected flow time (ccFT) percent change. **E** The optimal carotid artery maximum VTI threshold for distinguishing ≥  + 10% SV_∆_. Each data point represents a 10-s average. Prior to subsampling, there were 3596 data points categorized as <  + 10% SV_∆_ and 598 data points categorized as ≥  + 10% SV_∆_. The data categorized as <  + 10% SV_∆_ were randomly subsampled, iteratively 1000 times, to 598 data points (see methods). The sensitivity of maximum VTI is 532/598 = 89% and specificity is 598/598 = 100%. **F** The optimal ccFT threshold for distinguishing + 10% SV_∆_. Each data point represents a 10-s average. The sensitivity is 532/598 = 89% and specificity is 598/598 = 100%

The median and interquartile ranges for age and BMI were 27 (23—38) years and 23 (21.9–25.6) kg/m^2^, respectively; 39% were female. Vital signs and their change are previously reported [6]. Figure 2 A-D illustrates the progression of mean arterial pressure (MAP), SV, maximum VTI, ccFT and SV for all protocols. The optimal thresholds of % VTI_∆_, % ccFT_∆_ and their calculation are shown in Fig. 2 E and F. Both + 18% VTI_∆_ and + 4% ccFT_∆_ were 89% sensitive and 100% specific at detecting ≥  + 10% SV_∆._ The areas under their receiver operator curves were 0.97 and 0.98, respectively. Though not illustrated, a + 20% change in the *centroid* VTI had identical diagnostic characteristics.

### Discussion

In this model of hemorrhage and transfusion, the % VTI_∆_ and % ccFT_∆_ thresholds for detecting a ≥  + 10% SV_∆_ are consistent with earlier proof-of-concept data using a separate paradigm, also in healthy volunteers [9, 10]. With simulated blood transfusion, the 4% ccFT_∆_ approximates an absolute ccFT_∆_ of 10 ms, comparable to the 7 ms threshold observed in critically-ill patients with undifferentiated shock [4]. The + 18% VTI_∆_ threshold in this investigation is consistent with the slope of the SV_∆_- VTI_∆_ regression line, described previously [8]. Curiously, the + 20% threshold for the power-weighted (i.e., centroid) VTI matches the threshold of changing carotid blood flow in septic patients [3]. Assuming static vessel diameter, changing *centroid* velocity best approximates volumetric flow change [8].

### Limitations

Our findings have a number of limitations deserving of elaboration. First, from a broad hemodynamic perspective, common carotid blood flow relates to cardiac output (i.e., total blood flow) by the following, general relationship:

$$carotid \ blood \ flow \ = \ cardiac \ output \ x \ \frac{{Z_{whole\, body} }}{{Z_{carotid\, artery} }}$$

where *Z* is the impedance to flow for the listed vascular beds. Thus, using the carotid artery as a surrogate for SV_∆_ implies a constant ratio of whole body-to-downstream carotid impedance. That HR increased and MAP remained constant during the protocol, Z_whole body_ (e.g., total vascular resistance) likely rose relative to Z_carotid artery_, especially if downstream internal carotid artery impedance fell by auto-regulation. This could partly explain the relatively large change in carotid VTI (i.e., 18%) observed to detect a 10% SV_∆_. Second, though we did not measure carotid diameter to calculate carotid artery flow, the normal carotid artery pressure-diameter relationship is relatively flat above a MAP of 80 mmHg [11, 12]. More specifically, the carotid diameter changes by only 0.2 mm when MAP rises from 80 to 110 mmHg [12]; the average MAP for our subjects during the lowest tolerated LBNP stage was 99 mmHg. Nevertheless, even a 0.2 mm diameter change in a 7 mm carotid artery affects total flow by ± 6% [13]. On the other hand, in hypotensive patients, diameter assessment is likely more important; for example, measuring the diameter of the descending aorta improves the sensitivity of Doppler ultrasonographic flow assessment by approximately one-third in the critically-ill [14]. Third, we chose non-invasive pulse contour analysis as a gold standard because it is continuous, user-independent and accurately trends % SV_∆_, which is important for functional hemodynamic monitoring. However, absolute SV measures by non-invasive pulse contour analysis are less adequate, especially in the critically-ill [15, 16]. Still, we believe that the pattern of SV_∆_ that we observed is valid because it replicates the SV_∆_ measured in other LBNP investigations [17, 18] and is consistent with the LBNP SV_∆_ measured by other gold standards including left ventricular outflow tract VTI [19], bioimpedance [20] and bioreactance [21].

In summary, in this large data set of continuously-monitored SV synchronous with carotid Doppler, multiple measures from a wearable ultrasound patch identified + 10% SV_∆_ with high accuracy.

## Data Availability

The datasets used and/or analysed during the current study are available from the corresponding author on reasonable request.

## Abbreviations

AUROC: Area under the receiver operator curve
SV: Stroke volume
SV_∆_: Stroke volume change
VTI: Velocity time integral
ccFT: Carotid corrected flow time
VTI_∆_: Velocity time integral change
ccFT_∆_: Carotid corrected flow time change
FDA: Food and Drug Administration
LBNP: Lower body negative pressure
BMI: Body mass index
MAP: Mean arterial pressure
ms: Milliseconds
Z_whole body_: Whole body impedance
Z_carotidy artery_: Carotid artery impedance
mmHg: Millimeters of mercury
